# Discovery and characterization of long intergenic non-coding RNAs (lincRNA) module biomarkers in prostate cancer: an integrative analysis of RNA-Seq data

**DOI:** 10.1186/1471-2164-16-S7-S3

**Published:** 2015-06-11

**Authors:** Weirong Cui, Yulan Qian, Xiaoke Zhou, Yuxin Lin, Junfeng Jiang, Jiajia Chen, Zhongming Zhao, Bairong Shen

**Affiliations:** 1 Center for Systems Biology, Soochow University, Suzhou 215006, China; 2 Clinical Pharmacology Laboratory, the First Affiliated Hospital of Soochow University, Suzhou 215006, China; 3 School of Chemistry, Biology and Material Engineering, Suzhou University of Science and Technology, Suzhou 215009, China; 4 Department of Biomedical Informatics, Vanderbilt University School of Medicine, Nashville, TN, USA; 5 Department of Cancer Biology, Vanderbilt University School of Medicine, Nashville, TN, USA

## Abstract

**Background:**

Prostate cancer (PCa) is a leading cause of cancer-related death of men worldwide. There is an urgent need to develop novel biomarkers for PCa prognosis and diagnosis in the post prostate-specific antigen era. Long intergenic noncoding RNAs (lincRNAs) play essential roles in many physiological processes and can serve as alternative biomarkers for prostate cancer, but there has been no systematic investigation of lincRNAs in PCa yet.

**Results:**

Nine lincRNA co-expression modules were identified from PCa RNA-Seq data. The association between the principle component of each module and the PCa phenotype was examined by calculating the Pearson's correlation coefficients. Three modules (M1, M3, and M5) were found associated with PCa. Two modules (M3 and M5) were significantly enriched with lincRNAs, and one of them, M3, may be used as a lincRNA module-biomarker for PCa diagnosis. This module includes seven essential lincRNAs: TCONS_l2_00001418, TCONS_l2_00008237, TCONS_l2_00011130, TCONS_l2_00013175, TCONS_l2_00022611, TCONS_l2_00022670 and linc-PXN-1. The clustering analysis and microRNA enrichment analysis further confirmed our findings.

**Conclusion:**

The correlation between lincRNAs and protein-coding genes is helpful for further exploration of functional mechanisms of lincRNAs in PCa. This study provides some important insights into the roles of lincRNAs in PCa and suggests a few lincRNAs as candidate biomarkers for PCa diagnosis and prognosis.

## Background

Prostate cancer (PCa) is one of the most common types of cancer and is the second leading cause of cancer death in American men. The incidence of prostate cancer is increasing but varies remarkably among races and countries [1-4]. It has become fundamentally important to uncover the underlying mechanisms in prostate cancer due to the high risk of metastasis. Recent high throughput technologies, such as whole genome [5] and whole exome sequencing [6], have helped investigators to reveal genetic alternations including DNA structural change in the PCa genome. Epigenetic modification (e.g. DNA methylation, chromatin acetylation) also contributes to PCa development. For example, hypermethylation of CpG islands located in gene promoters (e.g., E-cadherin, PTEN, and RB) is frequently found in advanced PCa. Therefore, cancer is now considered to be a disease of the genome [7].

During the past decade, researchers have primarily focused on investigating the roles of protein-coding genes in cancer development. The recently published ENCODE project unveiled that a large portion (80.4%) of the human genome participates in at least one biochemical chromatin and/or RNA associated event in cells. Only about 2 percent of the genome is translated into proteins while the remaining is expressed as noncoding RNAs (ncRNAs) [8]. Noncoding RNAs have long been considered "junk RNA" or "transcriptional noise." In the world of ncRNAs, long non-coding RNAs (lncRNAs) are defined by the size >200 nt. Recently, it has been recognized that lncRNAs are a new class of ncRNAs for their essential roles in controlling every level of gene expression in various physiological processes, including development, differentiation and other biological mechanisms [9]. LncRNAs are considered one of the driving forces during tumorigenesis [10]. LncRNAs often overlap with or are interspersed between coding and non-coding transcripts. From the genetic point of view, lncRNAs can be classified into five broad categories [11]: (i) sense - when a lncRNA overlaps one or more exons of another transcript on the same strand, (ii) antisense - when a lncRNA overlaps one or more exons of another transcript on the opposite strand, (iii) bidirectional - when the expression of the lncRNA and a neighboring coding transcript on the opposite strand is initiated in close genomic proximity, (iv) intronic - when a lncRNA is derived from an intron of a second transcript, and (v) intergenic - when a lncRNA lies as an independent unit within the genomic interval between two genes. In this study, we used the RNA-Seq data of matched normal and tumor samples of PCa for studying the functions of long intergenic non-coding RNAs (lincRNAs). We focused on the co-expression between coding genes and lincRNAs to investigate the role of lincRNAs and to identify the putative lincRNA module biomarkers in prostate cancer, the PCa biomarker identification is becoming very essential in the era of post prostate-specific antigen [12,13].

## Methods

### Datasets

RNA-Seq data were downloaded from the National Center for Biotechnology Information (NCBI) Sequence Read Archive (SRA) [14] database with accession number SRP002628. This data includes the sequenced transcriptome (polyA+) of 20 prostate cancer tumors and 10 matched normal tissues using Illumina GAII platform. Bowtie2 index of the human genome (hg19) is retrieved from Bowtie2 [15] software website. The latest human Ensembl [16] annotation file (GRCh37) was downloaded from the Ensembl website. We obtained Gene Transfer Format (GTF) GENCODE Genes V17 Track file and lincRNA Transcripts Tracks file from the University of California Santa Cruz (UCSC) [17] Genome Browser Tables.

### Data preprocessing

We first used NCBI SRA toolkits from the NCBI website to transfer the sample data format from SRA to FASTQ. We used FastQC tool for checking quality of the sequencing data. Then, we wrote a Perl script to combine mRNA-lincRNA gene annotation files from UCSC Table Browser [17]. We removed the annotation that contained "exon," because the exonic structure is usually identified from the genomic alignment of the transcript sequence other than the protein sequence. Exons were numbered according to their positions within the mRNA sequence. "Exon" refers to transcription and "CDS" to translation, so we removed the exons and added lincRNA GTF files into a final file "hg19.mRNA.lincRNA.gtf" for TopHat mapping. We selected the gene names and Ensembl numbers which correspond to the gene names from the latest version of human Ensembl annotation file (GRCh37). Finally the corresponding relations of the two features were summarized in table "Gene-Ensembl" (Additional file 1 - Table S1) for conversion between gene symbol and EMSEMBL_gene_ID.

#### Pipeline for exploring lincRNA's functions in PCa

**Figure 1:**
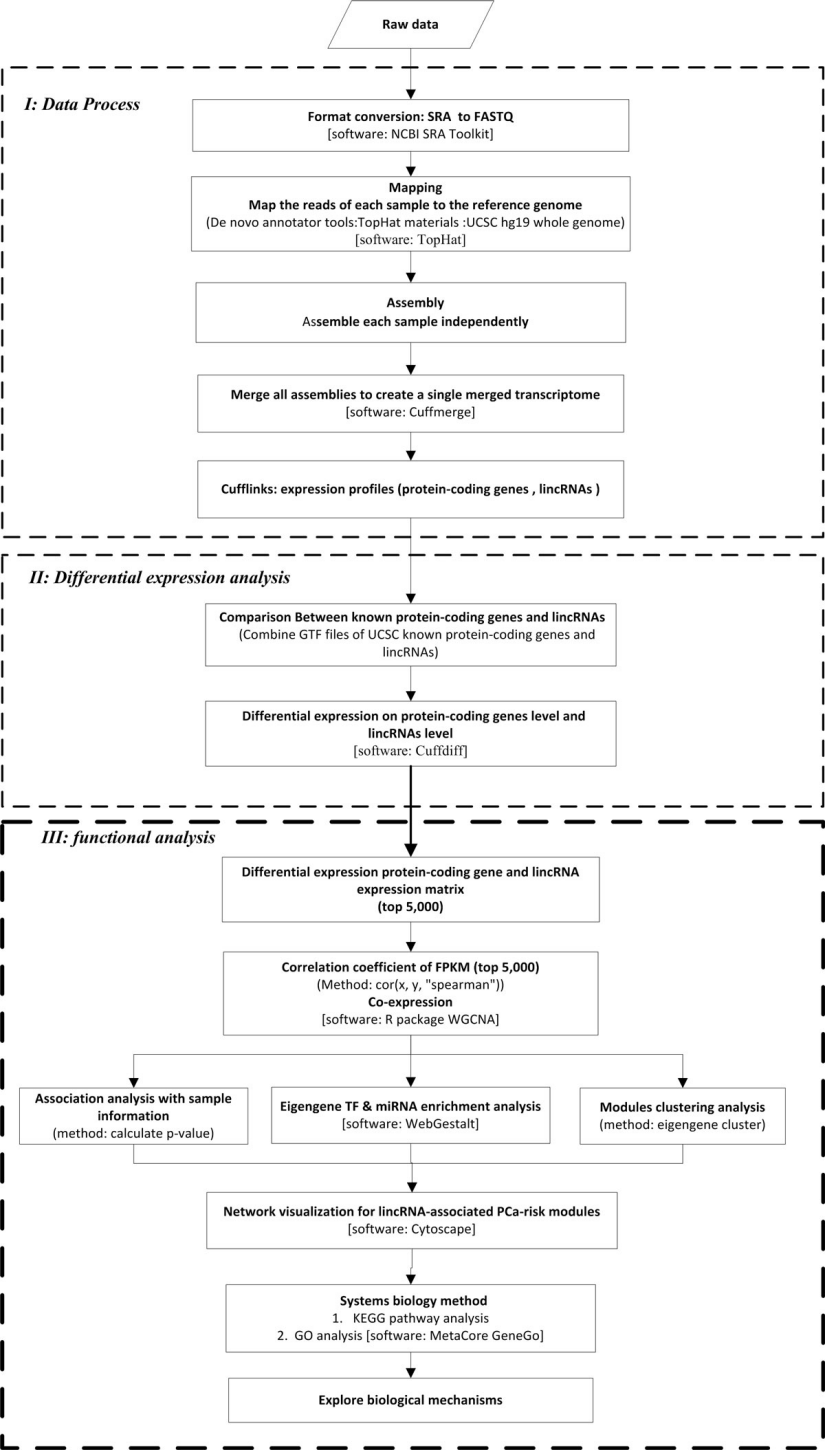
**Analysis pipeline for discovery and characterization of prostate cancer associated lincRNA modules and biomarkers**. (A) RNA-Seq data processing. (B) Differential expression analysis. (C) Functional analysis.

We explored the lincRNAs functions in the following 3 steps using RNA-Seq data. The detailed pipeline for data proves, differential gene expression and functional analyses is described in Figure 1.

#### Mapping, assembly and gene expression calculation

We used a *de novo *strategy to assemble the 30 samples and reconstruct the transcriptome. We used the TopHat 2.0.9 and Cufflinks 2.1.1 [18] for mapping and transcript assembly. Cufflinks adds the parameter "-G" in the step of assembly. We used the "hg19.mRNA.lincRNA.gtf" as GTF file and used it as a "reference" annotation.

#### Composite genes expression matrixes

We calculated the gene expression level only when the short reads could map to a gene. Expected fragments per 1000 base of transcript per million fragments mapped (FPKM) [19], has been typically used to measure genes expression level from RNA-Seq data. We wrote a Perl script to merge gene expression files from the 30 samples into a 30*116457 matrix file named "hg19.mRNA.lincRNAexprssion". Here, expression of each of those genes was calculated by Cufflinks. The first column of the matrix is the Ensembl number. Given that one gene may generate multiple transcripts due to alternative splicing, we removed the decimal points in the Ensembl numbers and chose the maximum FPKM value to represent the gene expression. Then, we mapped the Ensembl numbers to official gene names using "Gene-Ensembl" file. We finally obtained genes and lincRNA expression level in the whole set of tumor samples and the whole set of matched normal tissue samples, respectively.

#### Processing mRNAs and lincRNAs expression matrices

The expression matrix files were processed in the following four steps.

(1) Genes with null expression value in all 30 samples were removed.

(2) The non-zero minimum value of gene expression (the threshold set to 0.001) was assigned to replace zero values in the same line. For those genes that were not expressed in a sample, we assigned them the minimum value of the expression observed in other samples. The rationale is that these non-zero values represent minimum expression levels of genes and the transcripts are tissue-specific and under dynamic change, even though the expression level is very low. We did not remove the genes with zero expression values because they may represent weak signals.

(3) The rows with more than ten low expression genes (expression FPKM value < 0.05) were deleted.

(4) Finally, the expression matrix data was transformed by log2. The log2 transformation makes data convenient for gene expression comparison, as typically applied in gene expression studies.

#### Differential expression analysis (mRNAs and lincRNAs)

We used Cuffdiff [18] software to calculate the differential expression (DE) between tumor and tissue samples. First, we transformed the Ensembl number in the DE profile to the gene name based on the "gene-ensembl" file. For the same transcription, we chose the one with a smaller log2 fold-change value. P-values were calculated to evaluate the statistical significance of the differential expression and the cutoff was set as 0.05. The top 5000 differentially expressed genes were selected for further analysis (Additional file 2 - Table S2).

#### Network construction and module detection based on the differentially expressed genes

**Figure 2:**
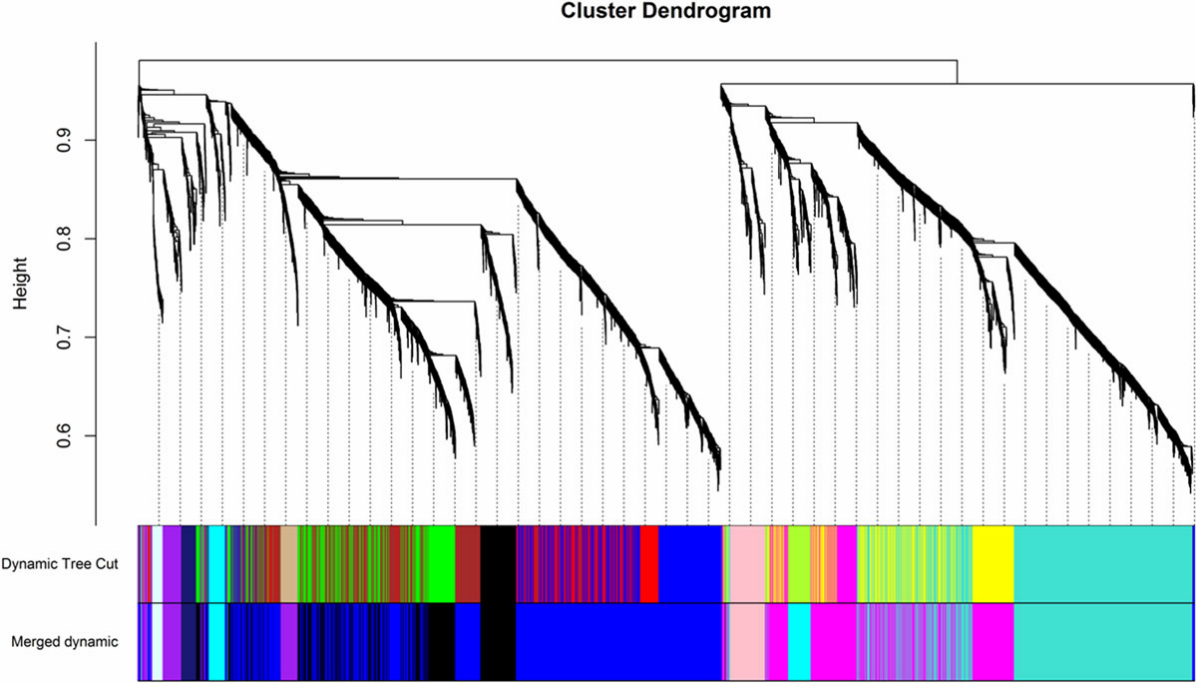
**Network construction and module identification**.

The network approach allows us to explore a set of interacting genes measured by modules or sub-networks that are involved in a complex disease like PCa. Gene co-expression analysis attempts to study the combined effects by identifying group of genes that are concordantly expressed, which may unveil the underlying molecular mechanisms of a disease [20]. For instance, Horvath and colleagues have developed a widely used algorithm WGCNA (weighted gene co-expression network analysis) [21] to search for co-expression modules. The R package WGCNA implements a suite of tools for network construction. The initial co-expression network based on Pearson's correlation coefficients may not be a scale-free network. In order to construct a scale-free network and identify important modules, we used a weighted adjacency matrix implemented in WGCNA. A step-by-step network construction and module detection method was used in our study. A selected power (power = 7) was determined through a soft-threshold approach implemented in WGCNA. With the constructed network, we then clustered the highly co-expressed genes into 9 co-expression modules (M1-M9). The clustering could be visualized in Figure 2, with each clustered module having a different color. The list of lincRNAs in each module is provided in Table 1.

**Table 1:** Summary of 9 co-expression network modules.

Module	# DE-PCGs*	# DE-lincRNAs*	Total genes	lincRNAs (%)
M1(Black)	630	23	653	3.50
M2(Blue)	1658	48	1706	2.80
M3(Cyan)	167	17	184	9.20
M4(Light cyan)	55	0	55	0
M5(Magenta)	740	84	824	10.19
M6(Midnight blue)	72	0	72	0
M7(Pink)	164	1	165	0.60
M8(Purple)	200	2	202	0.99
M9(Turquoise)	1105	34	1139	2.99
All in 9 modules	4791	209	5000	0.0418

*# DE-PCGs/lincRNAs: number of differentially expressed protein-coding genes or lincRNAs

#### Enrichment analysis of co-expression genes and identification of transcription factors or microRNAs associated with the modules

We performed canonical pathway analysis and Gene Ontology (GO) [22] analysis of the co-expression genes in the modules, both of which are commonly used in gene set enrichment analysis for understanding the functions of a set of genes. An integrated software suite, MetaCore™, was used for mapping the co-expression genes in the modules to functional categories. Significantly enriched pathways (p-value <0.01) from the MetaCore™ database were retrieved. For the 9 candidate modules, we recalculated the Module Membership (KME) of each gene by its correlation with module eigengenes. The online tool WebGestalt [23] was used to identify the module associated TFs and miRNAs. The identification is based on the enrichment and association analysis of miRNAs/TFs and their targeted genes[24].

## Results and discussion

### Overview of lincRNAs-mRNAs differential expression

As we described in the Methods section, we used RNA-Seq data from 20 PCa samples and 10 matched control samples to identify differentially expressed lincRNAs in PCa. The top 5000 differential raw expression files contained 209 lincRNAs and 4791 protein-coding genes. These 209 lincRNAs were mapped to the catalog of the Human Body Map lincRNAs [25] and 94 of them had corresponding transcript_id in the latest version of the annotation file. The list of these lncRNAs was provided in Additional file 3 - Table S3.

### Identification and characterization of PCa-associated co-expression modules

Co-expression modules were defined by a robust dynamic hierarchical tree and sets of tightly co-regulated genes with the measurement of dissimilarity (i.e. 1-topological overlap matrix) [26]. We set the minimum module size to 30 to ensure a qualified number of genes for the further analysis. The adjacent modules were merged based on the parameter of cutHeight. Modules with a minimum cutHeight 0.25 were merged. Principle component analysis (PCA) of the expression matrix for each module was performed. We denoted the first principal component (PC) as the module eigengenes and used it to represent the overall expression profile of the module [27]. We investigated the association between the PC of each module and the PCa phenotype by calculating Pearson's correlation coefficients. The p-value cutoff for the relationship was set to be 0.03. Three modules, M3 (*p *value = 0.028), M5 (*p *value = 1.49 × 10^-3^) and M1 (*p *value = 8.22 × 10^-3^) were found to be potential risk-related modules.

**Figure 3:**
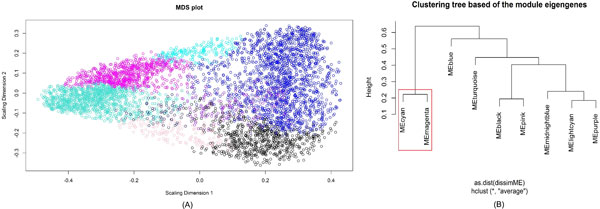
**Module eigengenes cluster analysis**. (A). Multi-dimensional scaling plots of the genes in the nine modules (M1: Black, M2: Blue, M3: Cyan, M4: Light cyan, M5: Magenta, M6: Midnight blue, M7: Pink, M8: Purple, M9: Turquoise). (B) Eigengene dendrogram.

**Figure 4:**
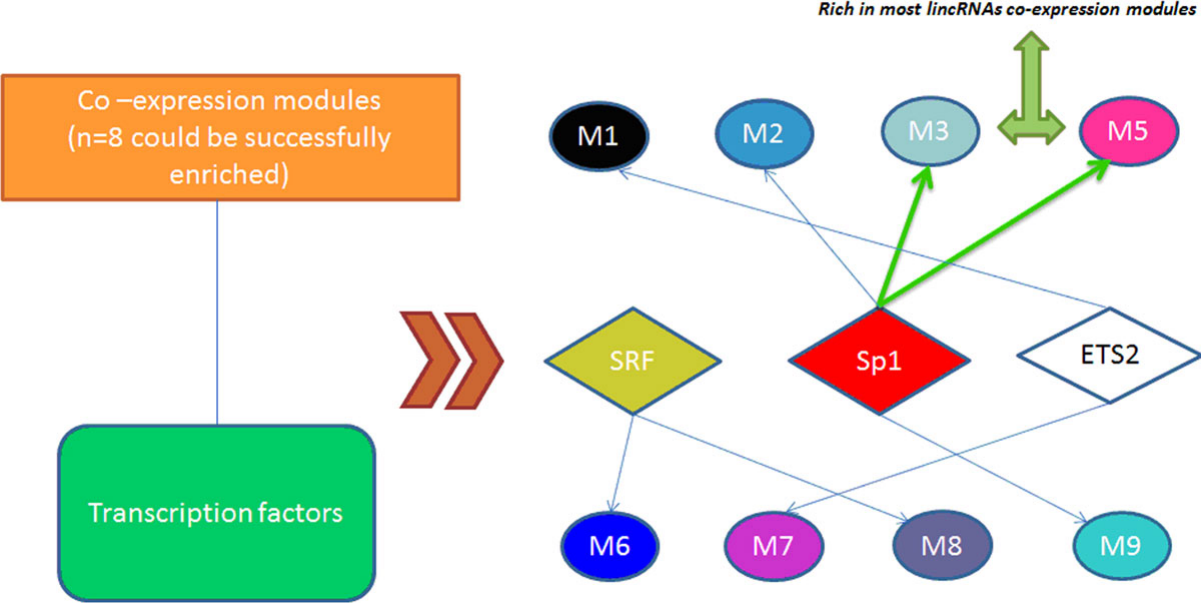
**Transcription Factor Enrichment analysis**. Sp1 was identified as an important regulator in 4 PCa associated co-expression modules.

The clustering analysis showed that the eigengenes of module M3 and module M5 are close to each other (M3: MEcyan, M5: MEmagenta in Figure 3). In addition, the eigengenes of these two modules are rich in lincRNAs and regulated by the same transcription factor Sp1 (Specificity protein 1) (see Figure 4), confirming the regulatory role of lincRNA in prostate cancer.

For each module gene, the KME value was calculated based on the correlation between the gene expression and the module eigengenes. The genes having the top 50 kME values in each module were used for the further analysis. These genes were provided in Additional file 4 - Table S4.

Eight of the nine modules were found significantly enriched with transcription factor and microRNA targets. Among them, three transcription factors, Sp1, SRF (serum response factor) and ETS2 (v-ets avian erythroblastosis virus E26 oncogene homolog 2) (Figure 4), and seven microRNAs, miR-200b, miR-15a, miR-24, miR-330, miR-17-5p, miR-155 and miR-101, are particular interesting because of the following two reasons. First, the value of adjP is smaller than 0.05 during the enrich process, which means that they have statistical significance. Second, these seven microRNAs have been reported to have potential regulatory roles in PCa (details in Additional file 5 - Table S5).

An indirect mechanism of androgen action has recently been identified in which Serum Response Factor (SRF) mediates the effects of AR (androgen receptor) on prostate cancer cells. Androgen-responsive SRF target genes affect the progression of PCa cell behavior by modulating cell migration, which may have implications for therapeutic intervention downstream of AR and SRF [28]. Likewise, the ETS2 was reported to be associated with PCa too. The presence of ETS2 is positively correlated with a more transformed phenotype and blockage of ETS2 function reduces transformed properties of prostate cancer cells [29]. Sp1 is an important transcription factor in various cellular processes and has been shown to be related to many types of tumorigenesis including prostate cancer [13,30]. Sp1 activates genes by binding to GC/GT-box sequences present within the gene's promoter region [31]. This activation leads to two glutamine-rich trans-activation domains which directly associate with the TATA-binding protein and the TBP-associated factor 4 [32]. Sp1 directly binds to histone acetyltransferase (CBP/p300) and recruits the ATP-dependent chromatin remodeling complex (SWI/SNF) [33,34]. According to the related study, Sp1 regulates key genes associated with PCa including androgen receptor (AR), c-Met, FAS, MMP, FLIP and TGF-β. It is clear that Sp1 plays an important role in the development of PCa, and our finding based on lincRNA module analysis suggested the Sp1 role may be acted through lncRNA too.

Interestingly, these modules are also enriched with microRNAs that are directly involved in the occurrence and progress of cancer. We conducted a literature survey and summarized a list of the reported microRNA-based biomarkers in PCa. Some of the module-enriched microRNAs are well-known biomarkers fro the PCa. For instance, miR-200B is a downstream target of androgen receptor which links its expression to decreased tumorigenicity and metastatic capacity of the prostate cancer cells [35]. Recent research shows that miR-24 could be an effective drug target for treatment of hormone-insensitive prostate cancer or other types of cancers [36]. miR-330 acts as an anti-metastatic miRNA in prostate cancer [37] and putative tumor suppressors. In addition, miR-15a is homozygously deleted in a subset of prostate cancers, suggesting that miR-15a could be important in the development of prostate cancer [38].

Our observation indicated that genes with similar functions within the same modules could contribute risk to prostate cancer in a co-expression manner. The modules with different functions can be regulated synergistically by the same genetic components, e.g. transcription factors and microRNAs, which play important roles in the development of prostate cancer. The enrichment analysis results of 8 modules revealed significant correlation between the modules.

The number of lincRNA in M3 and M5 modules is relatively high. Both of these two modules are associated with prostate cancer according to the p values, and they are regulated by the same genetic composition as Sp1. These observations indicated that lincRNAs may play roles as transcriptional regulators in prostate cancer.

### Characterization of lincRNAs in the M3 module

**Figure 5:**
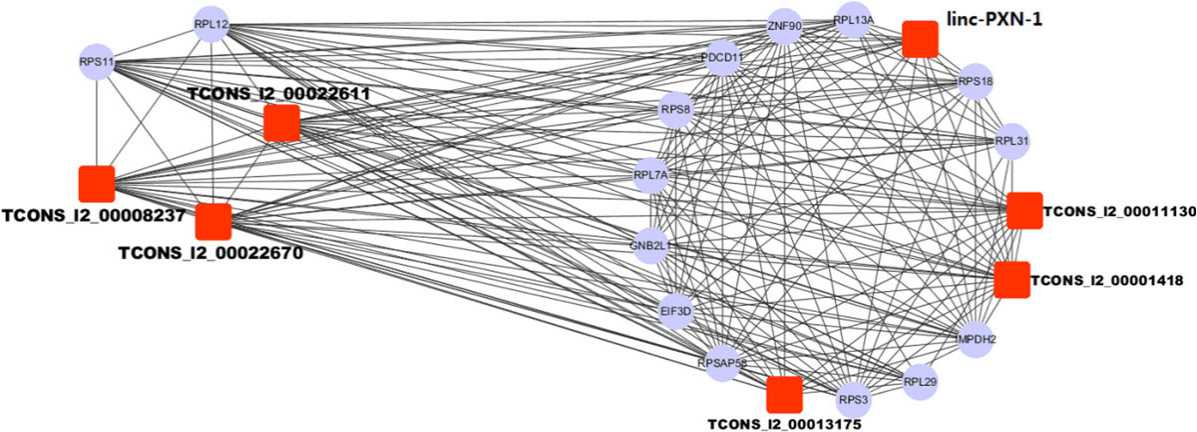
The M3 module is enriched with lincRNA hubs in the co-expression network.

Among the two modules enriched with lincRNAs (M3 and M5), M3 is particularly interesting regarding its association with PCa since it contains lincRNAs with top kME value (topGeneskME) while M5 module does not. We plotted the lincRNAs based co-expression module by its kME values and correlations (Figure 5). It is clear that this module is enriched with hub lincRNAs. This co-expression module contains 17 lincRNAs and 4 lincRNAs in topGeneskMElist; they are:"TCONS_l2_00011130", "TCONS_l2_00022611","TCONS_l2_00008516", and "TCONS_l2_00026666." According to the catalogue of the Human Body Map lincRNAs and TUCP transcripts [25], these four lincRNAs all belong to the TUCP (Transcripts of uncertain coding potential) catalogue. We then performed the Gene Set Enrichment Analysis (GSEA) of those genes in module M3. The Gene Ontology analysis revealed that this module was involved in "viral transcription" [39], "translation termination" [40], "viral gene expression" [41], "SRP-dependent cotranslational protein targeting to membrane" [42], and "cotranslational protein targeting to membrane". Furthermore, the genetic components regulate results showed that the M3 module was enriched with prostate cancer associated transcription factor Sp1, and Sp1 has been considered as an important target since it regulates important genes like androgen receptor (AR), c-Met, prostate-specific antigen (PSA) and transforming growth factor (TGF-β), etc., which are involved in cell cycle, proliferation, cell differentiation and apoptosis [43].

Five microRNAs were found to be significantly enriched in the M3 module. They are miR-24, miR-323, miR-518C, miR-149, and miR-96. miR-24 has been reported to have its function in prostate cancer development. We conducted the network analysis on the M3 module. We calculated the degree of the nodes in the M3 module network, according to the attribute of the network hubs. After filtering the low degree nodes (degree minimum value = 3), we used Cytoscape [44] for the network visualization. Finally, 7 lincRNAs (TCONS_l2_00022611, TCONS_l2_00008237, TCONS_l2_00022670, linc-PXN-1, TCONS_l2_00011130, TCONS_l2_00001418, and TCONS_l2_00013175; listed in Table 2) in the M3 module are included in this network for their regulatory function.

**Table 2:** LincRNAs (minimum degree value = 3) identified in M3 network analysis.

lincRNA	Name	Betweenness centrality	Network degree
TCONS_00020953	linc-PXN-1	9.90E-04	19
TCONS_l2_00008516	TCONS_l2_00008516	0	5
TCONS_l2_00022491	TCONS_l2_00022491	0	5
TCONS_l2_00013175	TCONS_l2_00013175	7.53E-05	14
TCONS_00019546	TSS14790	5.36E-05	3
TCONS_l2_00026666	TCONS_l2_00026666	0	3
TCONS_l2_00022670	TCONS_l2_00022670	1.18E-03	24
TCONS_l2_00001418	TCONS_l2_00001418	2.98 E-03	28
TCONS_l2_00022611	TCONS_l2_00022611	6.91 E-03	28
TCONS_l2_00012874	TCONS_l2_00012874	6.19E-05	13
TCONS_l2_00011130	TCONS_l2_00011130	3.26E-03	21
TCONS_l2_00008237	TCONS_l2_00008237	9.15 E-02	31
TCONS_00012018	linc-MLLT4-1	0	4

In summary, both the enrichment analysis and network analysis could validate the functional role of lincRNAs in prostate cancer. The results of enrichment analysis confirmed that this lincRNA-based co-expression module, M3, is biologically important in PCa. The lincRNAs in the M3 module might regulate protein-coding genes through transcription factors and/or microRNAs, and their abnormal changes may lead to prostate cancer development.

## Conclusions

Studies using high-throughput data have demonstrated that lincRNAs play roles in complex diseases including prostate cancer; however, the specific regulation has largely been unknown. In this study, we proposed a pipeline for discovery and characterization of prostate cancer associated lincRNA modules and biomarkers. A gene co-expression network was constructed using the whole transcriptome data that includes both lincRNAs and protein-coding genes. Through co-expression network analysis, we revealed 9 candidate modules that were differentially expressed between tumors and controls. The enrichment and association analysis with TF and microRNA highlighted the genetic factors that regulate the expression of the modules in a synergistic manner. This study helps to understand the potential functions and regulations of lincRNAs in prostate cancer, and also facilitates the development of diagnostic and prognostic tools for prostate cancer. The lncRNA analysis pipeline can also be applied to other complex disease studies including other types of cancer. The further experimental validation of the key TFs, microRNAs and the lincRNA module biomarkers in PCa will be our next step.

## Competing interests

The authors declare that they have no competing interests.

## Authors' contributions

WC and YQ carried out the differential expression analysis, participated in the network construction and drafted the manuscript. XZ, YL, JF and JC participated in the functional enrichment analysis and performed the statistical analysis. BS and ZZ conceived of the study, participated in its design and coordination and modified the manuscript. All authors read and approved the final manuscript.

## Supplementary Material

Additional file 1 **This table is for gene symbol and ENSEMBL_gene_ID conversion**. Click here for file

Additional file 2 **This table lists the top 5000 WGNA differentially expressed genes**. Click here for file

Additional file 3 **This table lists 94 annotated lincRNAs in the 209 differentially expressed lincRNAs in prostate cancer**. Click here for file

Additional file 4 **This table lists the genes with top 50 kME values in each module**. Click here for file

Additional file 5 **This table lists genetic components in the nine PCa associated modules**. Click here for file

## Declarations

The publication costs for this article were funded by the above grants.

This article has been published as part of *BMC Genomics *Volume 16 Supplement 7, 2015: Selected articles from The International Conference on Intelligent Biology and Medicine (ICIBM) 2014: Genomics. The full contents of the supplement are available online at http://www.biomedcentral.com/bmcgenomics/supplements/16/S7.
